# A High-Throughput, High-Containment Human Primary Epithelial Airway Organ-on-Chip Platform for SARS-CoV-2 Therapeutic Screening

**DOI:** 10.3390/cells12222639

**Published:** 2023-11-16

**Authors:** Christine R. Fisher, Felix Mba Medie, Rebeccah J. Luu, Robert B. Gaibler, Thomas J. Mulhern, Caitlin R. Miller, Chelsea J. Zhang, Logan D. Rubio, Elizabeth E. Marr, Vidhya Vijayakumar, Elizabeth P. Gabriel, Landys Lopez Quezada, Chun-Hui Zhang, Karen S. Anderson, William L. Jorgensen, Jehan W. Alladina, Benjamin D. Medoff, Jeffrey T. Borenstein, Ashley L. Gard

**Affiliations:** 1 Bioengineering Division, Draper, Cambridge, MA 02139, USA; crfisher@draper.com (C.R.F.); fmedie@draper.com (F.M.M.); rluu@draper.com (R.J.L.); rgaibler@draper.com (R.B.G.); tmulhern@draper.com (T.J.M.); vvijayakumar@draper.com (V.V.); egabriel@draper.com (E.P.G.); llopezquezada@draper.com (L.L.Q.); jborenstein@draper.com (J.T.B.); 2 Department of Chemistry, Yale University, New Haven, CT 06520, USAwilliam.jorgensen@yale.edu (W.L.J.); 3 Department of Pharmacology, Yale University, New Haven, CT 06520, USA; karen.anderson@yale.edu; 4 Department of Molecular Biophysics and Biochemistry, Yale University, New Haven, CT 06520, USA; 5 Division of Pulmonary and Critical Care Medicine, Massachusetts General Hospital, Boston, MA 02114, USA; jalladina@mgh.harvard.edu (J.W.A.); bmedoff@mgh.harvard.edu (B.D.M.)

**Keywords:** SARS-CoV-2, COVID-19, organ model, microfluidics, respiratory, coronavirus, epithelial cells, pandemic, throughput, antiviral

## Abstract

COVID-19 emerged as a worldwide pandemic in early 2020, and while the rapid development of safe and efficacious vaccines stands as an extraordinary achievement, the identification of effective therapeutics has been less successful. This process has been limited in part by a lack of human-relevant preclinical models compatible with therapeutic screening on the native virus, which requires a high-containment environment. Here, we report SARS-CoV-2 infection and robust viral replication in PREDICT96-ALI, a high-throughput, human primary cell-based organ-on-chip platform. We evaluate unique infection kinetic profiles across lung tissue from three human donors by immunofluorescence, RT-qPCR, and plaque assays over a 6-day infection period. Enabled by the 96 devices/plate throughput of PREDICT96-ALI, we also investigate the efficacy of Remdesivir and MPro61 in a proof-of-concept antiviral study. Both compounds exhibit an antiviral effect against SARS-CoV-2 in the platform. This demonstration of SARS-CoV-2 infection and antiviral dosing in a high-throughput organ-on-chip platform presents a critical capability for disease modeling and therapeutic screening applications in a human physiology-relevant in vitro system.

## 1. Introduction

Shortly after the appearance of the SARS-CoV-2 virus, with its high-rate of transmission and lack of cross-immunity in the human population [1], COVID-19 emerged as a global pandemic and it persists today. The deployment of multiple safe and efficacious vaccines [2] in a matter of months represents an extraordinary achievement built upon decades of mRNA technology development. However, the development of effective treatments for COVID-19, either from re-purposed drugs or new therapeutic candidates, has been far slower and beset by issues. For example, molnupiravir (Lagevrio) and the drug combination nirmatrelvir/ritonavir (Paxlovid) are the only two oral antiviral treatments available for COVID-19. Their emergency use authorization in the Unites States was not granted until December 2021, nearly two years into the pandemic, and treatment with each carries important caveats and adverse drug events. Researchers have raised concerns about the potential of the ribonucleoside molnupiravir to have mutagenic activity in the host cells [3]. The protease inhibitor Nirmatrelvir/ritonavir has achieved dramatic reductions in hospitalizations among at-risk populations but is less effective in healthier populations [4] and now carries an advisory of potential symptom rebound after usage [5]. Additional options for antiviral treatments are needed but may similarly falter without improved pre-clinical assessment. A major factor limiting drug development is the lack of pre-clinical screening systems that faithfully recapitulate key aspects of respiratory viral infections.

Immortalized cell culture platforms and animal models are standard tools for drug discovery, development, and preclinical research. Cell lines such as Vero E6 and A549 are frequently invoked for SARS-CoV-2 studies [6,7,8,9], but lack aspects of the host biology including ciliary differentiation and mucociliary clearance, both of which are believed to be critical for understanding pathogenesis and predicting therapeutic efficacy [10]. Significant progress in generating animal models for COVID-19 research has been reported [11,12], but availability was scarce early in the pandemic and animal models are inherently limited by their low throughput and imperfect correlation to clinical outcomes in humans. While not unique to COVID-19, these shortfalls represent particularly severe limitations in the context of a pandemic. Additionally, current antiviral drug development tools do not assess differences between individual patients in their vulnerability to severe infections and response to treatment.

We posit that several key characteristics could serve to build upon existing tools for SARS-CoV-2 therapeutic screening: (1) use of human primary cells, preferably drawn from a range of donors; (2) organ-on-chip technology to confer physiological cues and enable real-time in situ sensing; (3) a high-throughput platform compatible with pharmaceutical workflows; and (4) capable of implementation in a high-containment BioSafety Level 3 (BSL-3) to enable experiments with the native virus.

There are abundant examples of systems which successfully employ one or some of these characteristics but have limits as drug-screening tools. For example, studies using primary cells and SARS-CoV-2 are often seeded into membrane insert-based systems such as Transwells™ [8,13,14,15,16,17]. While primary tissue is superior to immortalized cell lines in biological fidelity, Transwells™ are limited by their poor usability, large media volume requirements, incompatibility with high-resolution imaging, and static growth conditions.

To improve upon these pitfalls, organ-on-chip technologies are designed to closely mimic not only the relevant tissue cell biology but other key aspects of the in vivo airway microenvironment, including the establishment of a fully differentiated pseudostratified epithelium with ciliary differentiation and beating, and mucus production. Some lung-on-a-chip systems seeded with human primary cells have been used for viral entry studies, but rely on pseudoviruses which lack a full viral life cycle and many potential antiviral targets [18,19]. Conversely, lung-on-a-chip devices have been seeded with immortalized cells and infected with native virus [20,21], but these systems lack key aspects of the physiological relevance of human primary airway cell culture at an air–liquid interface (ALI) [22]. Finally, work combining primary cells and wild-type virus in an organ-on-chip format has exhibited viral replication too low for rigorous drug development [23,24]. In addition, most organ-on-chip systems are low-throughput and require substantial expertise to operate.

**Figure 1:**
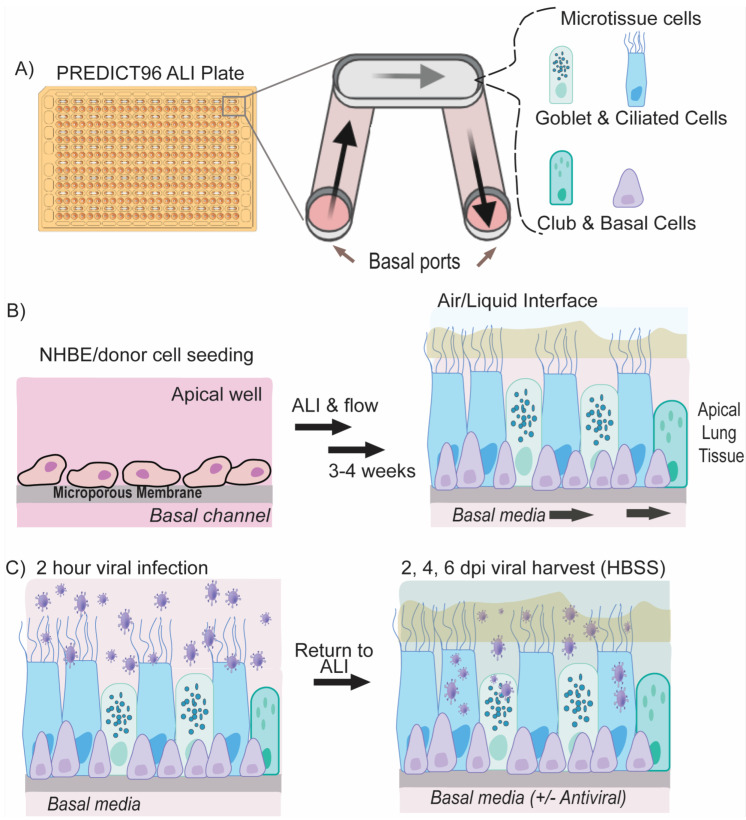
PREDICT96-ALI lung model and SARS-CoV-2 Infection. (**A**) PREDICT96-ALI system consists of a customized 96-well culture plate and a corresponding microfluidic pump system that can independently drive media flow on the apical well and/or the underlying basal channel. The apical well contains the differentiated airway tissue at the air–liquid interface while the PREDICT96 pump system recirculates media via the basal inlet and outlet port. (**B**) Normal human bronchial epithelial cells or small airway epithelial cells collected from human donors are seeded on the membrane of the apical well. After an initial submerged proliferation and differentiation phase, ALI is induced on the apical well where cells are further matured for 3–4 weeks with circulating custom ALI-media in the basal channel. (**C**) Mature tissue is infected with SARS-CoV-2 infection for 2 h; unbound viruses are removed and ALI is reintroduced. Virus are harvested at 2, 4, and 6 days post infection (dpi). After 6 days of infection, tissue can be fixed and imaged or processed for other downstream applications.

Here, we present the first report of a lung-on-chip platform operating in BSL-3 and supporting robust SARS-CoV-2 infection. This proof-of-concept COVID-19 therapeutic study employs several key improvements over traditional drug testing: The PREDICT96-ALI platform (Figure 1) uses (1) primary human airway epithelial cells from multiple donors grown in an (2) organ-on-chip format with active media perfusion situated in a (3) high-throughput, 96-well format which can be (4) operated in a high-containment BSL-3 environment and thus infected with native SARS-CoV-2. Tracheobronchial epithelial tissues from three human donors supported high titers of wild-type SARS-CoV-2 in this platform over 6-day infection periods. We further demonstrate that PREDICT96-ALI can model blunting of SARS-CoV-2 infection by two different drug classes: (1) the FDA-approved drug remdesivir, a SARS-CoV-2 RNA-dependent RNA polymerase inhibitor, and (2) Mpro-61, a non-covalent SARS-CoV-2 protease inhibitor, in a proof-of-concept assessment of antiviral efficacy. Mpro-61 was developed through computational and structure-based design and selected because of its promising drug-like properties, lack of off-target effects, and potency [25,26]. The PREDICT96-ALI platform has previously been demonstrated as a suitable system for modeling the infection of human primary airway epithelial cells with influenza virus and the human coronaviruses (HCoV) NL63 and OC43 [27]. The compact footprint and scalable form factor of the platform, when further adapted to downstream assays, have the potential to bring a streamlined, large-scale evaluation of therapeutic efficacy in specific patient populations to preclinical development. Altogether, PREDICT96-ALI offers the fidelity and relevance of a human primary cell-based system with the throughput of the cell line.

## 2. Materials and Methods

### 2.1. Microfluidic Platform and Integrated Micropumps

The PREDICT96 organ-on-chip platform consists of a microfluidic culture plate with 96 individual devices and a perfusion system driven by 192 microfluidic pumps integrated into the plate lid [28] (Figure 1A). As described in our previous work [27,28,29,30], in order to establish recirculating flow in the microchannels, we incorporate a self-priming micropump array into the lid that serves as the fluidic interface with the culture plate via stainless steel tubes. The PREDICT96 pumping system has 192 pneumatically-actuated micropumps embedded in the plate lid: two per culture device, one for the chamber above the membrane and one for the microchannel below. In the PREDICT96-ALI platform, since the upper chamber is at the air–liquid interface (ALI), only the 96 pumps serving the microchannel are in use during experiments. The actuation of the micropumps transfers media between the inlet and outlet wells of the bottom microfluidic channel, establishing a hydrostatic pressure differential, which results in flow through each microchannel.

### 2.2. Preparation of Human Primary Bronchial Epithelial Cells from Healthy Living Donors

Human small airway epithelial cells were isolated from two living donors by research bronchoscopy at Massachusetts General Hospital (MGH), Boston, MA, USA. Bronchoscopy brushings were collected into collection media (RMPI (Thermo Fisher, Waltham, MA, USA) + 2% human serum albumin (Thermo Fisher) + 5 µM ROCKi (Tocris, Minneapolis, MN, USA) and transported on ice to Draper (Cambridge, MA, USA) [31]. All research involving human subjects was approved by the MGH and Partners HealthCare System (Mass General Brigham, Boston, MA, USA) Institutional Review Board (protocol number 2015P000319 and date of approval 05/05/2015), and it adhered to all applicable institutional and sponsor ethical guidelines, including but not limited to the anonymization of donors for personal identity. All methods were in adherence with applicable safety and laboratory practice guidelines. Normal human bronchial epithelial cells (NHBEs) from an additional cadaver donor were purchased from Lifeline Cell Technology, Frederick, MD, USA (catalog number FC-0035, lot number 04401).

### 2.3. Culture, Cryopreservation, and PREDICT96-ALI Seeding and Differentiation of NHBEs

Living donor cells reported in this manuscript were derived from donor DH01 (28 years, female, Asian (non-Hispanic)) and donor DH02 (52 years, male, White (non-Hispanic)). Cells were cultured in SAGM + 4i media [27], and passaged using Accutase (Sigma, St. Louis, MO, USA). After 4 passages, cells were cryopreserved in Cryostor 10 (STEMCELL Technologies, Cambridge, MA, USA).

The commercially sourced primary human normal bronchial epithelial cells (Lifeline Cell Technology) reported in this manuscript were derived from donor 04401 (16 years, male, Caucasian). Cells were cultured in Bronchialife Epithelial Airway Medium (Lifeline Cell Technology), and passaged using Accutase (Sigma). After 4 passages, cells were cryopreserved as described above.

PREDICT96-ALI plates were prepared as previously described [27] (Figure 1B). Living donor-derived cells were thawed and cultured to confluency prior to introduction into PREDICT96-ALI plates. Cells were seeded at 15,000 cells per device in a 3 µL volume directly onto the membrane. Commercially sourced cells were thawed and plated at 10,000 cells per device in a 3 µL volume directly onto the membrane. Cell/tissue growth occurred for 48 h in submerged proliferation media (SAGM + 4i: SAGM (Lonza, Lexington, MA, USA), 100 U/mL penicillin–streptomycin (Thermo Fisher), 5 μM ROCKi (Tocris), 1 μM A-83-01 (Tocris), 0.2 μM DMH-1 (Tocris), and 0.5 μM CHIR99021 (Tocris)), followed by the initiation of differentiation using 48 h of submerged differentiation media (HBTEC-ALI, Lifeline Cell Technology, plus 100 U/mL penicillin/streptomycin) and the subsequent induction of an ALI at day 5 of culture, as previously reported, using either custom-ALI media [27] or Pneumacult-ALI media (STEMCELL Technologies). Tissues were matured over the course of 4 weeks in ALI culture with active pumping prior to viral infection experiments. To remove accumulated mucus from the apical surface of the maturing tissue, 100 µL of 1× Hank’s Balanced Salt Solution (HBSS, Sigma) was added to the apical surface of each tissue and incubated on the tissues for 1 h at 37 °C, subsequently followed by an additional 5 min wash with 100 µL of 1× HBSS at room temperature every 7 days (d).

### 2.4. Immunofluorescence and Confocal Imaging

Immunofluorescence (IF) staining was conducted as previously reported [27]. Primary antibodies were used to stain basal cells (CK5, Thermo Fisher), goblet cells (Muc5A, Thermo Fisher), ciliated cells (acetylated-tubulin, Abcam, Waltham, MA, USA) and SARS-CoV-2 (spike and nucleocapsid proteins, GeneTex, Irvine, CA, USA). The SARS-CoV-2 nucleocapsid protein antibody was used alone or in combination with an antibody against SARS-CoV-2 spike. The secondary antibodies used were Alexa Fluor 488 goat anti-mouse IgG (Thermo Fisher) and Alexa Fluor 555 goat anti-rabbit IgG (Thermo Fisher). Tissues were incubated with counter-stains Hoechst 33342 dsDNA nuclear stain (Thermo Fisher) and Phalloidin-iFluor 647 reagent (Abcam). Antibody-labeled PREDICT96-ALI tissues were imaged in situ within the PREDICT96-ALI plate by way of the optical qualities of the PREDICT96-ALI platform and by using a Zeiss (White Plains, NY, USA) LSM700 laser scanning confocal microscope and Zen 2012 SP5 FP3 (Black) software version 14.0.17.201 (Zeiss). Tile scans and z-stacks of the tissues at 40× and 10× magnification were acquired.

### 2.5. Mean Fluorescence Intensity (MFI) Quantification

Tile scans (10×) of the middle of each PREDICT96-ALI tissue device were captured on a Zeiss LSM700 laser scanning confocal microscope with Zen 2012 SP5 FP3 (Black) software version 14.0.17.201 (Zeiss) at 16 bit per pixel. The MFI of the signal from the red channel, or the SARS-CoV-2 nucleocapsid or spike protein expression, of these regions was measured via ImageJ Fiji version 2.15.0. The average signal from the secondary-only controls was subtracted to remove the background signal, and the average MFI from each infection condition was calculated.

### 2.6. Club-Cell Secretory Protein (CCSP) Luminex

After 4 weeks of ALI culture of DH01 human primary airway epithelial cells, basal media samples were collected for the quantification of soluble CCSP via a premade Magnetic Luminex Performance Assay kit (Uteroglobin/SCGB1A1, R&D Systems, Minneapolis, MN, USA). Culture media were collected from the bottom chamber of each tissue device and immediately frozen at −20 °C until use. Undiluted media samples were processed according to the manufacturer’s protocol and analyzed via an Invitrogen Luminex FLEXMAP 3D instrument and the analytics xPONENT software version 4.2 (Invitrogen, Waltham, MA, USA). The data collected were used to generate standard curves for each analyte using a five-parameter logistic (5-PL) curve fit to determine the concentration of CCSP in the sample. The concentration of CCSP was measured for each individual PREDICT96-ALI tissue device, and CCSP levels measured from devices derived from the same experimental conditions were averaged (*n* = 6).

### 2.7. Inoculation of PREDICT96-ALI Tissues with SARS-CoV-2

All of the work with SARS-CoV-2 was performed at BSL-3. SARS-CoV-2 strain USA-WA1/2020, passage 4 virus was obtained from Joseph Brewoo and Sam R. Telford III (New England Regional Biosafety Laboratory, Global Heath & Infectious Disease, Tufts University, North Grafton, MA, USA). A passage 5 stock was generated by propagating the virus on Vero E6 cells (ATCC, Manassas, Virginia, USA) in DMEM (Gibco, Waltham, MA USA) supplemented with 0.1% bovine serum albumin (Sigma), 1× penicillin/streptomycin (Gibco), 1× non-essential amino acids, NEAA (Gibco). Inoculation of PREDICT96-ALI airway tissues with SARS-CoV-2 (and mock control) was performed similarly to coronavirus infections described previously [27]. Briefly, the PREDICT96-ALI tissues were prepared by performing a mucous wash with HBSS. Freshly thawed SARS-CoV-2 was diluted in the same infection media used for viral propagation to various MOIs: 0 (mock control), 0.025, 0.25 or 2.5. Donor DH01 was inoculated with passage 4 virus while the other donors received passage 5 virus. Inoculum was incubated on the apical surface of the tissues for 2 h rocking at 34 °C. Inoculum was subsequently removed, and the apical side of each tissue device was washed 4× with HBSS. The final wash was reserved as a 0 days post-infection (d.p.i.) sample. Fresh custom-ALI or Pneumacult-ALI media were introduced into the basal ports of each tissue device before static incubation (no pumping) at 34 °C.

On 2, 4, and 6 d.p.i., the apical surface of the tissues was washed 2× with HBSS (40 min per wash). The two HBSS washes from each individual device were pooled and stored at −80 °C. Fresh custom-ALI or Pneumacult-ALI media were introduced into the bottom chamber before returning the plate to 34 °C. At the conclusion of the experiment on day 6 p.i., the PREDICT96-ALI airway tissues were fixed with 4% paraformaldehyde (Electron Microscopy Sciences, Hatfield, PA, USA) for 1 h at room temperature. Tissues were then washed with and incubated in phosphate-buffered saline (PBS) until staining.

### 2.8. Antiviral Dosing

Remdesivir (MedChemExpress, Monmouth Junction, NJ, USA) and Mpro-61 [26] were used in antiviral screens performed on PREDICT96-ALI tissue. Both compounds (or dimethyl sulfoxide (DMSO) alone as the vehicle) were diluted from stock concentrations to 10 µM in complete Pneumacult-ALI. Diluted antivirals or vehicle were applied to the basal channels of PREDICT96-ALI airway tissues 1 h after infection and on days 2 and 4 p.i.

### 2.9. RNA Extraction and RT-qPCR

Viral RNA was extracted from HBSS wash samples and tissue RNA was extracted from tissue devices as previously described [27]. Briefly, QIAamp Viral RNA Mini Kits (Qiagen, Beverly, MA, USA) and RNeasy Micro Kits (Qiagen) were used for RNA extraction following the manufacturer’s protocols. One-step reverse transcription quantitative polymerase chain reaction (RT-qPCR) was performed on extracted RNA samples using a QuantiTect Probe RT-PCR kit (Qiagen) with 7.8 μL purified viral RNA or specified quantities (2.5 ng or 100 ng) of tissue RNA in a 20 μL reaction. The assays were performed using an Applied Biosystems QuantStudio 7 Flex System (Thermo Fisher) using the following conditions: 50 °C for 30 m, 95 °C for 15 m, 45 cycles of 94 °C for 15 s, and 60 °C for 60 s. All primers and probes used in this study are summarized in Table S1. Absolute quantification (copies/mL) of SARS-CoV-2 N1 and/or N2, as well as RNase P control of wash samples and tissue RNA were calculated using a standard curve generated from serial dilutions of linearized SARS-CoV-2 viral RNA (American Type Culture Collection, ATCC). The quantification of tissue transcript expression was calculated using the comparative cycle threshold (Ct) values and the relative quantification method described by Schmittgen and Livak [32]. The comparative cycle threshold (Ct) values were normalized to the housekeeping gene GAPDH and to undifferentiated basal airway cells.

Washes from at least *n* = 4 (and up to *n* = 24) PREDICT96-ALI devices per condition per time-point were tested via RT-qPCR in duplicate. Samples with cycle threshold (ct) values above 37 were omitted. Absolute quantification of supernatant viral RNA was calculated using a standard curve generated from serial dilutions of 2019-nCoV_N_Positive Control (IDT, Coralville, IA, USA, 10006625).

### 2.10. Plaque Assay

Wash samples collected from inoculated PREDICT96-ALI plates were titered via plaque assay as previously reported [33]. Briefly, Vero E6 cells (ATCC) were plated in 6-well plates and incubated at 37 °C and 5% CO_2_ until confluent. For each condition, samples from 6 devices with the highest viral titers measured using RT-qPCR were selected for assessment by plaque assay. HBSS wash samples were diluted 1:5 or 1:10 initially and then in 10-fold serial dilutions in 300 μL total. After a PBS wash, Vero E6 cells were incubated with viral dilutions for 2 h at 37 °C, rocking every 15 min. Cells were then washed with PBS, overlaid with DMEM supplemented with final concentrations of 2% fetal bovine serum (FBS), 1× NEAA, 1× penicillin/streptomycin, and 1.2% cellulose (colloidal and microcrystalline (Sigma)), and incubated for 3 days. After the removal of the overlay, plates were washed with PBS, fixed with 4% PFA for 30 min, stained with a 0.1% crystal violet (Sigma)/4% ethanol solution, and imaged. Plaque-forming units/milliliter (PFU/mL) were calculated as follows: number of plaques/(dilution × total inoculum volume (mL)).

### 2.11. Statistical Analysis

Data are presented as mean ± standard deviation or standard error of the mean (indicated in figure legends) and were analyzed using Prism version 9.1.2 for Windows (GraphPad Software, Boston, MA, USA). Statistical significance was determined using either one- or two-way analysis of variance (ANOVA) with Dunnett’s test for multiple comparisons (indicated in figure legends). A *p*-value lower than 0.05 was considered statistically significant.

## 3. Results

### 3.1. Formation of Mature Human Airway Tissue from Research Bronchoscopy-Derived Donor Cells

**Figure 2:**
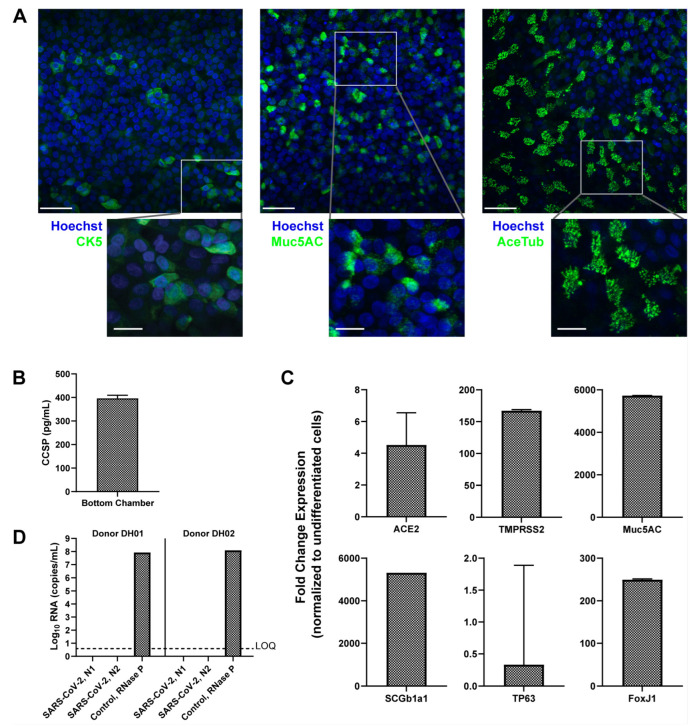
Donor cells derived from research bronchoscopy and differentiated in PREDICT96-ALI exhibit the structure and composition of the mature human airway. (**A**) Representative max projection immunofluorescence (IF) images demonstrating the presence of basal (CK5), goblet (Muc5AC), and ciliated (acetylated-tubulin or AceTub) cells in healthy, uninfected pseudostratified PREDICT96-ALI airway tissue derived from donor DH02 and grown for 4 weeks at air liquid interface (ALI), taken at 40× magnification and shown with a 50 µm scale bar. The inset images have a 20 µm scale bar. Tissues were counter-stained for dsDNA with Hoechst stain. (**B**) Club Cell Secretory Protein (CCSP) detection in PREDICT-ALI airway experiments containing donor DH01 at 4 weeks ALI. Media samples collected from the basal microfluidic chamber of each PREDICT96-ALI tissue exhibit detectable levels of CCSP (mean ± SEM of *n* = 6). Tissue media samples randomly selected from two independent, representative experiments. (**C**) Relative fold change in expression of mRNA transcripts from differentiated airway tissues in PREDICT96-ALI derived from donor DH01. Reverse transcription quantitative polymerase chain reaction (RT-qPCR) was used to detect transcripts, using GAPDH as a reference gene and normalizing it to undifferentiated cells (mean fold change expression ± SEM). *n* = 12 healthy tissue replicates selected at random from two representative PREDICT96-ALI experiments. (**D**) Confirmation that epithelial cells isolated from donors DH01 and DH02 are SARS-CoV-2 negative, as determined by RT-qPCR detecting two regions in the SARS-CoV-2 nucleocapsid (N) gene, N1 and N2. RNaseP probed as a tissue sample control. Limit of quantification (LOQ) indicates copy number corresponding to Ct values of ≥40 cycles. Samples that did not meet the minimum signal intensity (undetermined) after 40 PCR cycles are excluded. Data presented as mean Log10 viral RNA copies/mL ± SEM (*n* = 2).

Human tissues differentiated and maintained in PREDICT96-ALI were either derived from freshly harvested human epithelial cells from living research bronchoscopies, as previously reported [27], or purchased commercially. The airway tissues used in the current SARS-CoV-2 infection studies resembled previously reported [27] pseudostratified, 30–50 µm thick tissues matured over 4 weeks in the PREDICT96-ALI platform. In situ immunofluorescence (IF) staining of representative donor DH02 confirmed the relative populations of basal cells (CK5), goblet cells (Muc5AC) and ciliated cells (acetylated-tubulin) within the mature tissues demonstrating the cellular and functional heterogeneity of the human airway epithelium (Figure 2A). The tissues exhibited an expected proliferative basal cell layer immediately adjacent to the semi-permeable membrane of each tissue device, with the robust expression of mucin proteins indicating the presence of functional goblet cells throughout the tissue, and abundant distinct ciliary islands (associated with ciliated columnar epithelial cells) across the apical surface of the tissue, with relative quantities of each cell type matching prior reports [34,35,36,37]. Similarly, tissue function was monitored and club cell presence was determined by measuring club cell secretory protein, CCSP, and secretion into the media in the basal chamber of each device via Luminex from representative donor DH01 at 4 weeks of ALI (Figure 2B) [38]. These data demonstrate that primary human airway basal cells can be successfully implemented and matured in the PREDICT96-ALI platform to form tissue with composition, architecture and function representative of key features of the in vivo human airway.

To confirm tissue maturation, key gene transcripts associated with differentiated airways were measured in both undifferentiated and mature tissues from two representative donors (DH01 and 04401) and normalized to GAPDH: Muc5AC (goblet cells), SCGB1A1 (club cells), FoxJ1 (ciliated cells) and TP63 (basal cells) (Figure 2C). TP63 expression is notably reduced for differentiated tissue relative to undifferentiated tissue, an expected result as the mature human airway is composed of a variety of differentiated epithelial cell types and only a small percentage of self-renewing basal progenitor cells [36,39]. Expression levels of SARS-CoV-2 viral receptors were also established in mature uninfected tissues from the same donors: angiotensin-converting enzyme 2 (ACE2) and transmembrane protease serine 2 (TMPRSS2) (Figure 2C). Finally, we confirmed that donors DH01 and DH02 were negative for SARS-CoV-2 transcripts prior to in vitro infection: neither had measurable levels of SARS-CoV-2 N1 or N2 viral proteins but robust quantities of RNase P transcripts were detected as a control (Figure 2D). The commercially sourced tissue was collected well before SARS-CoV-2 circulation and was therefore not tested. Altogether, the PREDICT96-ALI tissues were in a mature, differentiated state with representative cell surface features required for SARS-CoV-2 infection studies and were negative for SARS-CoV-2 prior to inoculation at 4 weeks of ALI.

### 3.2. PREDICT96 Airway Tissue Supports SARS-CoV-2 Replication

**Figure 3:**
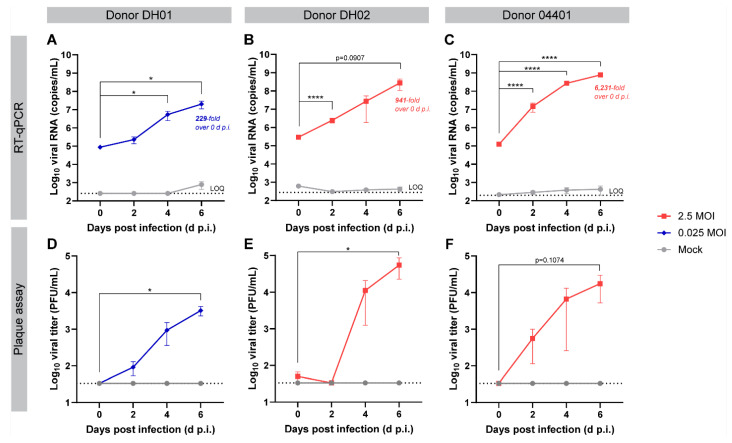
SARS-CoV-2-inoculated PREDICT96-ALI airway tissues from multiple donors yield replicative virus by RT-qPCR and plaque assay. (**A**–**C**) RT-qPCR analyses for SARS-CoV-2 nucleoprotein RNA copies in the apical wash of PREDICT96-ALI airway tissue at 0, 2, 4, and 6 days post infection (d.p.i.). Panels represent unique donors: (**A**) donor DH01 (*n* = 12 tissue replicates per time-point and condition); (**B**) donor DH02 (*n* = 12 tissue replicates for mock, *n* = 18 for 2.5 MOI); (**C**) donor 04401 (*n* = 12 tissue replicates for mock, *n* = 24 for 2.5 MOI). Fold increase in average RNA titer between days 0 and 6 indicated for each donor. An independent experiment with donor 04401 taken to 12 d.p.i. indicated that infection peaked at 6 d.p.i (Figure S1). LOQ (dotted line): Log10 viral RNA (copies/mL) corresponding to cycle threshold values ≥ 37. (**D**–**F**) Plaque assays detecting replicative virus from apical wash samples of PREDICT96-ALI airway tissue at 0, 2, 4, and 6 d.p.i. Panels represent unique donors: (**D**) donor DH01; (**E**) donor DH02; (**F**) donor 04401. *n* = 3 tissue replicates for mock-infected conditions; *n* = 6 tissue replicates per time-point in the infected condition, selected after exhibiting the highest viral RNA titers as determined by RT-qPCR. LOQ (dotted line): ≤33.3 PFU/mL. Samples for RT-qPCR and plaque assays came from the same experiments, except for donor 04401, in which RT-qPCR and plaque assay data came from independent experiments. SARS-CoV-2 (USA-WA1/2020) at passage 4 was used to infect the tissue in panels (**A**,**E**); passage 5 was used to infect all other panels. All data are shown as mean ± SEM. Statistical significance determined via a two-way analysis of variance (ANOVA) with Dunnett’s test for multiple comparisons (comparing to 0 d.p.i.): * *p* ≤ 0.05; **** *p* ≤ 0.0001.

To test if PREDICT96-ALI lung tissue could support robust viral replication, we inoculated mature airway tissue with SARS-CoV-2 (USA-WA1/2020 strain) (Figure 1C). SARS-CoV-2 nucleocapsid (N) RNA was detected in the apical washes from tissues infected with MOI 0.025 (donor DH01, Figure 3A) or MOI 2.5 (donors DH02 and 04401, Figure 3B,C, respectively). Over the course of a 6-day infection, viral RNA copy numbers increased to 229-, 941-, and 6231-fold in donors DH01, DH02, and 04401, respectively. These infection kinetics highlight the platform’s capacity to resolve donor-to-donor variability, though further mechanistic conclusions based on differences in tissue characterization are beyond the scope of this work. An independent experiment with donor 04401 taken to 12 d.p.i. indicated that infection peaked at 6 d.p.i. (Figure S1). Apical tissue wash samples were also assessed for live virus by a standard plaque assay (Figure 3D–F). Plaque-forming virus was recovered from infection in all three donors, with plaques generally observable from samples with >10^6^ viral RNA copies/mL.

### 3.3. SARS-CoV-2 Foci and Co-Localized Staining with Ciliated and Secretory Cells

**Figure 4:**
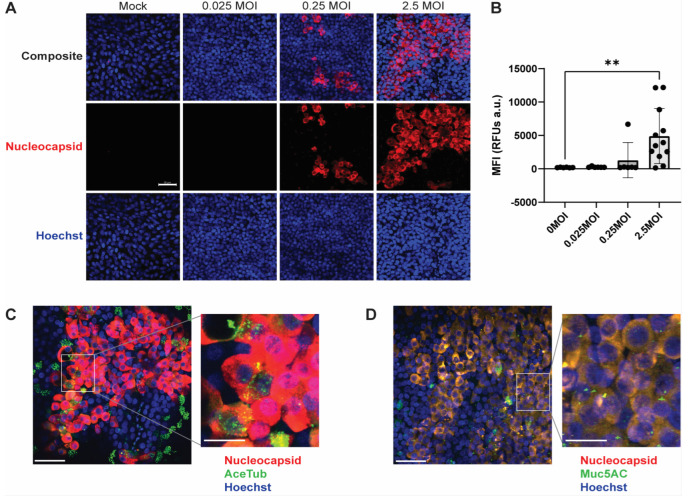
PREDICT96-ALI airway tissue exhibits heterogeneous SARS-CoV-2 viral foci and co-localized staining with differentiated airway epithelial cell types. (**A**) Max projection IF images depicting positive staining of the SARS-CoV-2 N (red) and dsDNA (blue) within PREDICT96-ALI airway tissue from donor 04401 (donor DH01 in Figure S2). Tissues were fixed with 4% paraformaldehyde at 6 d.p.i. following inoculation with SARS-CoV-2. Images are 40× magnification with a 50 µm scale bar. (**B**) Mean Fluorescent Intensity (MFI) image quantification of SARS-CoV-2 infection in PREDICT96-ALI airway tissues associated with panel (**A**). Images were taken at 10× magnification and analyzed for MFI using ImageJ Fiji. Statistical significance determined via a one-way ANOVA with Dunnett’s test for multiple comparisons (** *p* ≤ 0.01). *n* = 6 for mock, 0.025 and 0.25 MOI; *n* = 12 for 2.5 MOI. Data also correspond to fluorescence images in Figure S3. (**C**,**D**) Representative max projection IF image of SARS-CoV-2 N (red) and (**C**) ciliated cells (acetylated α-tubulin, green) or (**D**) goblet cells (MUC5AC, green) and dsDNA (blue) within PREDICT96-ALI airway tissue from donor 04401 at 6 d.p.i. following inoculation with SARS-CoV-2 at 2.5 MOI. Images were taken at 40× magnification and are shown with 50 µm scale bars. The insets have 20 µm scale bars.

SARS-CoV-2 infection in PREDICT96-ALI airway tissues was characterized by IF microscopy. Figure 4A shows max projection images of PREDICT96-ALI devices containing donor 04401 tissues inoculated with SARS-CoV-2. The images exhibit greater abundance of SARS-CoV-2 nucleocapsid protein (red) as MOI increases. Strong expression of nucleocapsid protein is seen at 6 d.p.i. within the 2.5 MOI-infected tissues. Multi-cell clusters indicate SARS-CoV-2 replication and spread to neighboring cells, an observation also reported in prior studies working with in vitro primary upper airway models [16]. MFI quantification of SARS-CoV-2 infection demonstrates a significant increase in the expression of SARS-CoV-2 positive cells at 2.5 MOI (Figure 4B). Similar data from lower MOI infections were observed in donor DH01 (Figure S3). Collectively, these data indicate robust virus infection and the ability to closely track infection kinetics.

Max projection 40× tile images depict SARS-CoV-2-infected PREDICT96-ALI tissue (red) counter-stained with acetylated tubulin (AceTub, green) for ciliated cells (Figure 4C) or with Muc5AC (green) for secretory goblet cells (Figure 4D). The images highlight heterogeneous SARS-CoV-2 viral foci and co-localization between SARS-CoV-2 staining to ciliated cells and goblet cells, indicating that these cell types are likely to target respiratory epithelial cell types of SARS-CoV-2, consistent with prior reports [40,41]. Cytopathic effect (CPE) is also observed in SARS-CoV-2-infected tissues, as indicated by the irregular, enlarged epithelial morphology observed in some SARS-CoV-2-positive cells (Figure 4C,D). Data showcasing the susceptibility of multiple lung cell types to SARS-CoV-2 infection are enabled by the compatibility of the platform with in situ IF staining and imaging.

### 3.4. Treatment with Antiviral Compounds from Two Drug Classes Inhibit Viral Replication

**Figure 5:**
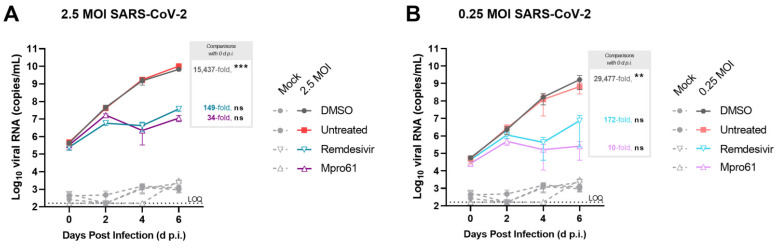
Assessment of viral replication in tissues from donor 04401 with and without antiviral interventions. Viral replication is measured by RT-qPCR for SARS-CoV-2 N copies in the apical wash of PREDICT96-ALI airway tissue from donor 04401 at 0, 2, 4, and 6 days post infection (d.p.i.). Remdesivir, Mpro-61, or DMSO (vehicle) were dosed at 10 μM into the basal media of tissues infected with SARS-CoV-2 1 h after infection and again on days 2 and 4 p.i. Tissues were infected with either 2.5 MOI (**A**) or 0.25 MOI (**B**). Data shown in mean ± SEM, and average fold-change over 0 dpi is noted. *N* = 4 per condition. Statistical significance determined by a two-way ANOVA with Dunnett’s test for multiple comparisons (comparing to 0 d.p.i.): ns *p* > 0.05; ** *p* ≤ 0.01; *** *p* ≤ 0.001.

As an initial feasibility demonstration, tissues from donor 04401 were treated with two antiviral drugs during SARS-CoV-2 infection. The nucleoside analog remdesivir and the protease inhibitor Mpro-61 [26] (Figure S4) were added at 10 μM to basal media 1 h after infection and again on days 2 and 4. Untreated tissue and tissue treated with the antiviral vehicle (DMSO) supported robust infection through 6 d.p.i., similar to the infection observed in Figure 2D and Figure S1. Treatment with either remdesivir or Mpro-61 reduced viral genome copy numbers occurred on days 4 and 6 p.i. Tissues treated with antivirals yielded significantly smaller viral load as shown by the fold increase between 0 and 6 d.p.i. These effects were observed at both 2.5 (Figure 5A) and 0.25 MOI (Figure 5B).

## 4. Discussion

Animal models and cell culture platforms are fundamental tools for modeling respiratory viruses and evaluating the efficacy of candidate therapeutics. However, they do not always accurately recapitulate human-specific mechanisms of viral infection and responses to antiviral therapies [10]. Organ-on-chip technologies have the potential to overcome these limitations by offering the physiological fidelity of human tissue in an in vitro assay format. Organ-on-chip platforms have historically been challenged by practical limitations such as excessive complexity, poor usability, low throughput and incompatibility with pharmaceutical laboratory workflows. Here, we report SARS-CoV-2 infection and antiviral efficacy in PREDICT96-ALI, a lung-on-chip platform for high-throughput primary cell culture at high containment, which overcomes the critical shortcomings of traditional organs-on-chip. To our knowledge, this manuscript serves as the only known demonstration of therapeutic screening with primary human airway tissue in an organ-on-chip model in a BSL-3 environment.

PREDICT96-ALI supports robust viral replication across multiple donors, as measured by three independent readout assays (RT-qPCR, plaque assay and immunofluorescence microscopy). For sample sets assayed by two or all three of these readouts, there was good agreement in data trends between assays. Viral copy numbers, measured by RT-qPCR, were comparable to prior reports in other in vitro primary airway models and in vivo clinical specimens (sputum, swab) from patients with active virus replication in tissues of the upper respiratory tract [40,42]. Similarly, infectious titer is comparable to prior reports in other in vitro primary airway models [13,16], further validating the model’s capacity to support SARS-CoV-2 replication. Although there are a small number of publications showcasing the use of human lung-on-chip platforms for modeling native SARS-CoV-2 infection (all comprising alveolar tissue), PREDICT96-ALI is the only organ-on-chip platform reported to successfully demonstrate robust viral replication in a human primary cell-based model of the tracheobronchial airway.

The isolation of human airway epithelial cells from living donors creates a pathway to study specific patient subpopulations of interest and characterize variability in SARS-CoV-2 infection and antiviral therapies. Even among a small number of donors, we observed variable expression levels of key lung cell markers (Figure 2) and variable infectability (Figure 3) which enables a future opportunity to investigate mechanistic insights within donor-to-donor variability. Furthermore, having demonstrated the ease of integrating multiple donor tissues and given its high-throughput nature, the model is positioned to accommodate larger suites of tissues to cover a broad range of physiologies.

To further demonstrate the utility of the PREDICT96-ALI platform, we performed a proof-of-concept study with the antiviral compounds remdesivir, an FDA-approved inhibitor of the SARS-CoV-2 RNA-dependent RNA polymerase, and Mpro-61, an experimental inhibitor of the SARS-CoV-2 main protease (Mpro) which cleaves viral polyproteins (Figure 5). The selection of Mpro-61 resulted from computational and structure-based design efforts to identify potent noncovalent Mpro inhibitors [25,26,43]. One aspect of the design involved the incorporation of drug-like properties into the molecules as part of lead optimization, including optimal absorption, distribution, metabolism, excretion (ADME) features and lack of toxicity. We previously used in vitro pharmacological profiling to examine possible off-target effects as well as antiviral efficacy for the inhibition SARS-CoV-2 in cell culture [25,26]. Mpro-61 was selected for further evaluation as one of our most promising compounds that had drug-like properties, a lack of off-target effects and antiviral potency. Both remdesivir and Mpro-61 reduced viral RNA washed from apical washes by nearly three orders of magnitude, a robust dynamic range that illustrates the utility of PREDICT96-ALI in discerning antiviral efficacy from distinct drug classes. Future studies can expand upon these pilot experiments to assess antiviral efficacy in more quantitative measurements (such as half maximal inhibitory concentration), with more recent SARS-CoV-2 isolates, and across donor populations.

Depending on experimental parameters, PREDICT96-ALI could be employed to evaluate therapeutic candidates at several points along the discovery, development, or preclinical stages of the pharmaceutical pipeline. PREDICT96-ALI could complement existing preclinical models by generating efficacy, toxicity, and dosing data to help select lead candidates for further study, or could eventually even supplant other in vitro or in vivo models. Furthermore, screening on human tissues from diverse backgrounds and with co-morbidities would safely garner data only obtainable after regulatory approval—an unprecedented capability.

One current limitation of this system is the time required to generate mature lung tissue (3–4 weeks). While this constraint is shared by all microphysiological systems, PREDICT96-ALI was designed for compatibility with standard laboratory automation equipment to reduce user touch time. Here, we have used largely manual methods as proofs-of-concept but anticipate future development, including that of tissue maintenance and downstream assays, to leverage liquid handling robotics and proceed similarly to assay development within standard 96-well plates.

Altogether, the results reported here demonstrate the application of the PREDICT96-ALI platform toward modeling SARS-CoV-2 infection at BSL-3 and the antiviral activity of compounds from two different drug classes in primary human airway tissue, tested across multiple donor cell populations and experimental conditions. PREDICT96-ALI provides the throughput, biological complexity, and model robustness necessary to rapidly evaluate a wide-range of infection parameters, observe critical insights into the pathogenesis of SARS-CoV-2, and screen potential therapeutics of potential potency.

## 5. Conclusions

In this work, we present the first report of a primary human cell-based lung-on-chip platform, known as PREDICT96-ALI, operating in a high-containment BSL-3 environment and supporting robust native SARS-CoV-2 infection. We demonstrate that the high-throughput (96× microtissue format) and high-fidelity PREDICT96-ALI platform can support native SARS-CoV-2 replication at physiologically relevant titers in tracheobronchial tissues derived from primary human airway epithelial cells from multiple donors over 6-day infection periods. Furthermore, we show that PREDICT96-ALI can model the attenuation of SARS-CoV-2 viral replication by antiviral compounds of different drug classes, remdesivir and Mpro-61. Altogether, this proof-of-concept COVID-19 therapeutic study illustrates the potential for microphysiological systems, specifically PREDICT96-ALI, to provide streamlined, preclinical evaluation of antiviral efficacy in diverse patient populations.

## Data Availability

All data will be available in the Microphysiological Systems Database, curated and maintained by the MPS for COVID Research (MPSCoRe) Working Group, NICEATM.

## Supplementary Materials

The following supporting information can be downloaded at https://www.mdpi.com/article/10.3390/cells12222639/s1, Table S1: Primers and probes used in RT-qPCR assays; Figure S1: Infection over twelve days; Figure S2: Immunofluorescence staining of SARS-CoV-2-inoculated PREDICT96-ALI airway tissues from donor DH01; Figure S3: Tile scan images; Figure S4: Antiviral compound structures.
