# Supplementary material for: A High-Throughput, High-Containment Human Primary Epithelial Airway Organ-on-Chip Platform for SARS-CoV-2 Therapeutic Screening
Source: Cells. 2023 Nov 16;12(22):2639. doi: 10.3390/cells12222639 (PMC10669988; doi:10.3390/cells12222639)
Supplement: Supplementary file 1 [file cells-12-02639-s001.zip › cells-2593807-supplementary.pdf]

Supplementary Materials for

A High-Throughput, High-Containment Human Primary Epithelial Airway Organ-on-Chip Platform for SARS-CoV-2 Therapeutic Screening

Christine R. Fisher†, Felix Mba Medie†, Rebeccah J. Luu†, Robert B. Gaibler, Thomas J. Mulhern, Caitlin R. Miller, Chelsea Zhang, Logan D. Rubio, Elizabeth E. Marr, Vidhya Vijayakumar, Elizabeth P. Gabriel, Landys Lopez Quezada, Chun-Hui Zhang, Karen S. Anderson, William L. Jorgensen, Jehan W. Alladina, Benjamin D. Medoff, Jeffrey T. Borenstein and Ashley L. Gard*

†Authors contributed equally
*Corresponding author. Email: agard@draper.com

This file includes:

Table S1
Figures S1 to S4

Primers, Probes and TaqMan® Assays	Vendor, Catalogue/Assay Number
nCOV_N1 Forward Primer	IDT, 10006830
nCOV_N1 Reverse Primer	IDT, 10006831
nCOV_N1 Probe	IDT, 10006832
nCOV_N2 Forward Primer	IDT, 10006833
nCOV_N2 Reverse Primer	IDT, 10006834
nCOV_N2 Probe	IDT, 10006835
RNase P Forward Primer	IDT, 10006836
RNase P Reverse Primer	IDT, 10006837
RNase P Probe	IDT, 10006838
ACE2 TaqMan Gene Expression Assay	Thermo Scientific, Hs01085333_m1
TMPRSS2 TaqMan Gene Expression Assay	Thermo Scientific, Hs01122322_m1
SCGB1A1 TaqMan Gene Expression Assay	Thermo Scientific, Hs00171092_m1
Muc5AC TaqMan Gene Expression Assay	Thermo Scientific, Hs01365616_m1
FoxJ1 TaqMan Gene Expression Assay	Thermo Scientific, Hs00230964_m1
TP63 TaqMan Gene Expression Assay	Thermo Scientific, Hs00978340_m1

Table S1.
Primers, probes and TaqMan® Assays used in RT-qPCR assays.

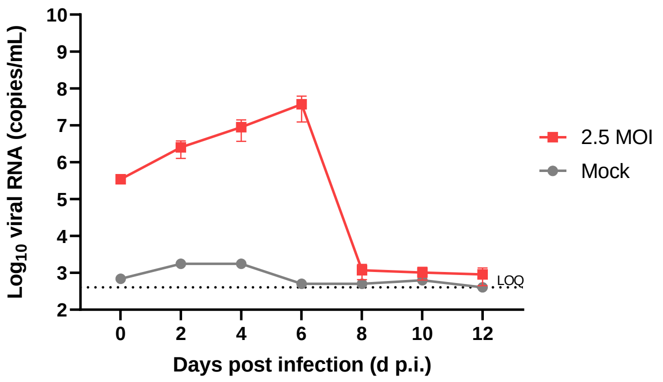

Figure S1.
Type RT-qPCR analyses for SARS-CoV-2 nucleoprotein RNA copies in the apical wash of PREDICT96-ALI airway tissue from donor 04401 (N = 6 tissue replicates for mock, N = 6 for 2.5 MOI) at 0, 2, 4, 6, 8, 10, and 12 days post infection (d p.i.). Infection peaked at 6 d p.i. Limit of quantification (LOQ, dotted line): Log10 viral RNA (copies/ml) corresponding to cycle threshold values ≥ 37..

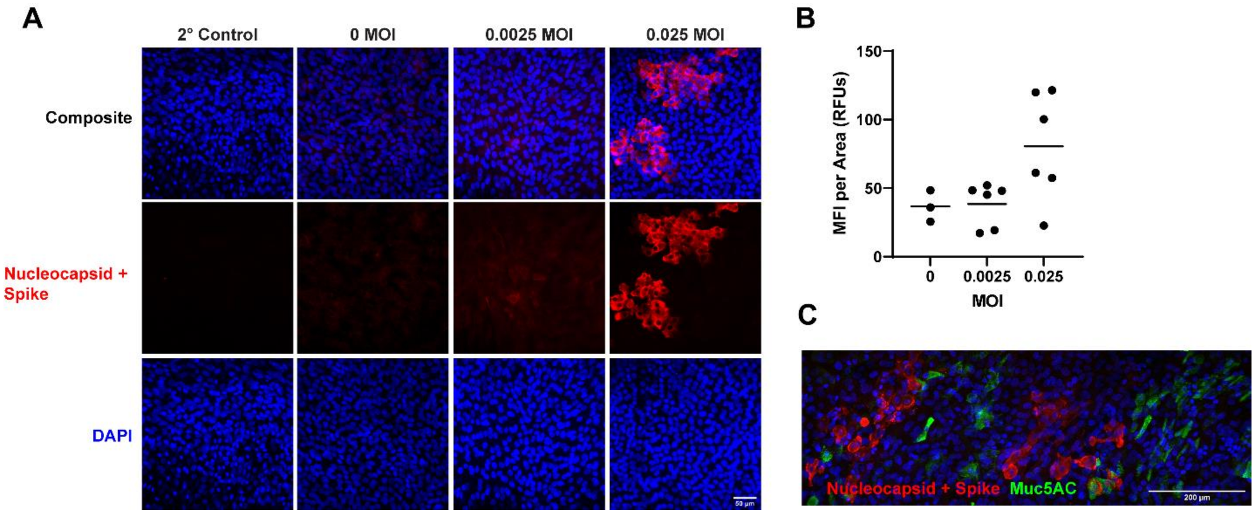

Figure S2.
Immunofluorescence staining of SARS-CoV-2-inoculated PREDICT96-ALI airway tissues from donor DH01. (A) Max projection immunofluorescence images depicting positive staining of the SARS-CoV-2 spike and nucleocapsid proteins (S and N, respectively, red) and dsDNA nucleic acids (Hoechst 33342, blue) within PREDICT96-ALI airway tissue from donor DH01. Tissues were fixed with 4% paraformaldehyde at 6 d p.i. following inoculation with SARS-CoV-2 USA-WA1/2020 (MOI 0, 0.0025 or 0.025). Secondary antibody control showed low background noise. Images were taken at 40x magnification and are shown with a 50 μm scale bar. One representative region of interest (ROI) from N = 1 tissue device shown per MOI. (B) Mean Fluorescent Intensity (MFI) image quantification of SARS-CoV-2 infection in PREDICT96-ALI airway tissues associated with panel A. MFI from the red channel (S and N proteins) indicate a trend of increased signal in the 0.025 MOI condition, but is not significant compared to the 0 or 0.0025 MOI conditions. The average MFI of the 0 (N = 3 tissue replicates), 0.0025 (N = 6 tissue replicates), and 0.025 MOI (N = 6 tissue replicates) are 36.67±11.45, 38.38±15.75, 80.52±39.69 RFUs (mean ± standard deviation), respectively. Images were taken at 10x magnification and analyzed for MFI using ImageJ Fiji. (C) Representative max projection immunofluorescence image of SARS-CoV-2 positivity (S and N proteins, red), goblet cells (Muc5AC, green) and dsDNA nucleic acids (Hoechst 33342, blue) within PREDICT96-ALI airway tissue from donor DH01 at 6 d.p.i. following inoculation with SARS-CoV-2 at 0.025 MOI. Image was taken at 40x magnification and is shown with a 200 μm scale bar.

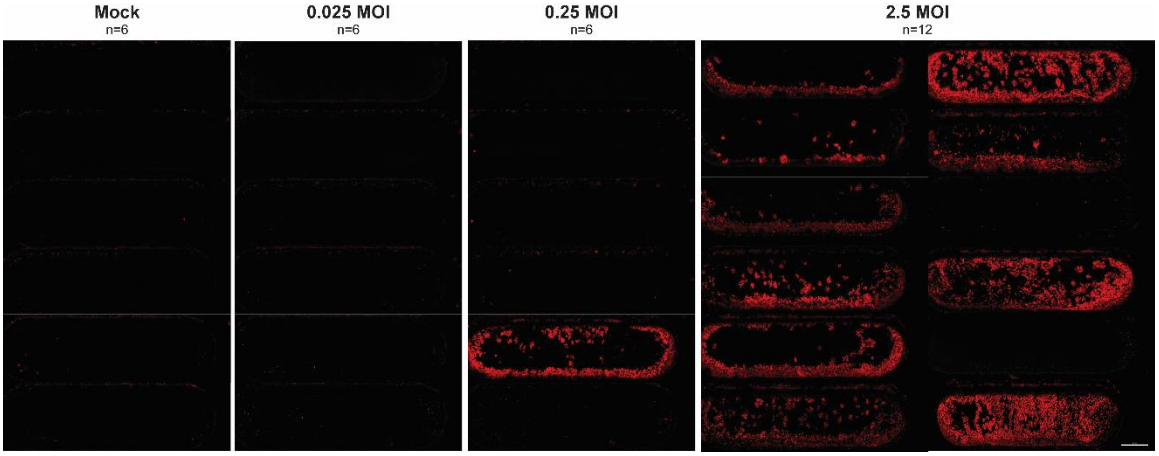

Figure S3.

Tile scan images depicting SARS-CoV-2 positivity (Nucleocapsid, red) in 30 devices of PREDICT96-ALI airway tissues from donor 04401 at 6 d p.i. following inoculation with SARS-CoV-2 at 0, 0.025, 0.25, and 2.5 MOI. Images were used for MFI quantification (see figure 3B). Images were taken at 10x magnification and are shown with a 500 µm scale bar.

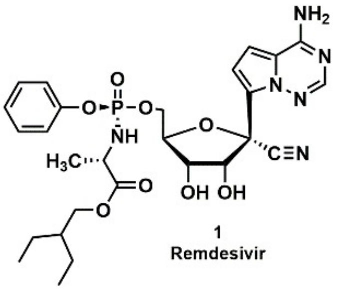

1
Remdesivir

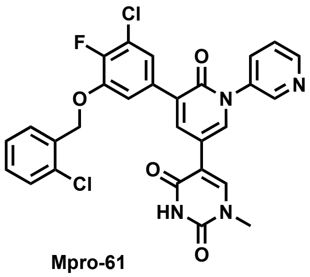

Mpro-61

Figure S4.
Antivirals evaluated in SARS-CoV-2-inoculated PREDICT96-ALI tissues. Remdesivir, a SARS-CoV-2 RNA-dependent RNA polymerase inhibitor (left) and Mpro-61, a SARS-CoV-2 protease inhibitor (right).
